# Supplementary material for: Bulk transparent supramolecular glass enabled by host–guest molecular recognition
Source: Nat Commun. 2024 May 9;15:3929. doi: 10.1038/s41467-024-48089-4 (PMC11082146; doi:10.1038/s41467-024-48089-4)
Supplement: Supplementary file 1 — Supplementary Information [file 41467_2024_48089_MOESM1_ESM.pdf]

Supplementary Information

Bulk Transparent Supramolecular Glass Enabled by Host–Guest Molecular Recognition

Changyong Cai[1], Shuanggen Wu[1], Yunfei Zhang[1], Fenfang Li[2], Zhijian Tan[3*] & Shengyi Dong[1*]

[1]*College of Chemistry and Chemical Engineering, Hunan University, Hunan 410082, P. R. China;*
[2]*College of Chemistry and Chemical Engineering, Central South University, Hunan 410083, P. R. China.*
[3]*Institute of Bast Fiber Crops, Chinese Academy of Agricultural Sciences, Hunan 410205, P. R. China;*

*To whom correspondence should be addressed.
E-mail: dongsy@hnu.edu.cn; tanzhijian@caas.cn

1. The property of **MH** 3
2. NMR spectra of **MH** 3
3. Molecular docking 3
4. Fourier-Transform IR (FT-IR) spectra of **MH** 4
5. Thermogravimetric Analysis (TGA) of **MH** 5
6. Broadband dielectric measurements of **MH** 5
7. Single crystal pattern of *para*-hydroxybenzoic acid 6
8. Low field nuclear magnetic resonance of **MH** 6
9. Molecular dynamic (MD) simulations 7
10. Rheological testing of **MH** 8
11. Powder X-ray diffraction (PXRD) patterns of **MH** 8
12. Small angle X-ray scattering (SAXS) of **MH** 9
13. Solid-phase ultraviolet spectrum of **MH** 9
14. Differential scanning calorimeter (DSC) of **MH** 10
15. Transmittance of **MH** 10
16. Refractive index measurement of **MH** 11
17. Mechanical testing of **MH** 12
18. Dynamic mechanic thermal analysis (DMA) of **MH** 12
19. Photos of **MH** with additives 12
20. Atomic force microscopy (AFM) of **MH** 13
21. Fluorescence of **MH** with additives 14
22. NMR spectra of reagent 16

1. The property of MH

Supplementary Tab. 1. Essential information of **MH**.

Host	Guest	Molar ratio	Density (g cm^{-3})	T_g (°C)
**M**	**H**	1:1	1.27	86.10

Supplementary Tab. 2. Molar ratio of glass formation.

M:H	1:0	10:1	5:1	5:2	2:1	1:1	1:2	1:4	1:8	1:10	0:1
Glass formation	No	No	No	Yes	Yes	Yes	Yes	No	No	No	No

2. NMR spectra of MH

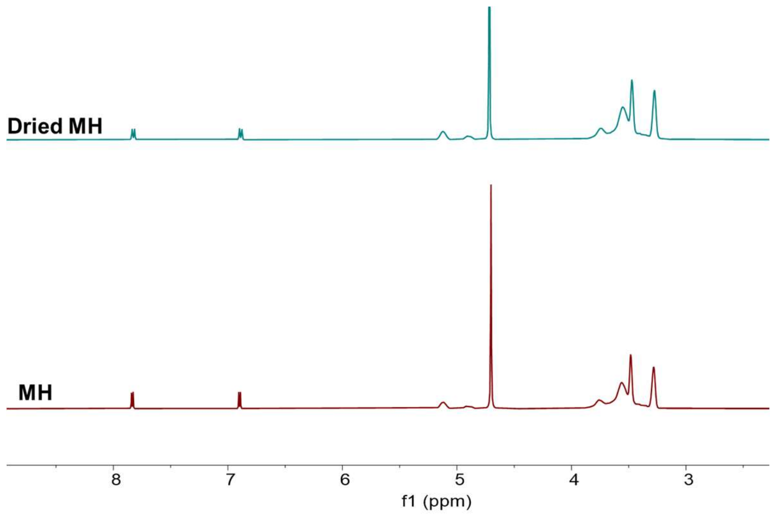

Supplementary Fig. 1. ^{1}H NMR spectra of freshly prepared **MH** and dried **MH** (400 MHz, D_2O, 25 °C).

As shown in this Supplementary Fig. 1, dried **MH** has the same NMR signals as those of freshly prepared **MH**. Thus, after drying under vacuum, there are non-covalent reactions between **M** and **H**.

3. Molecular docking

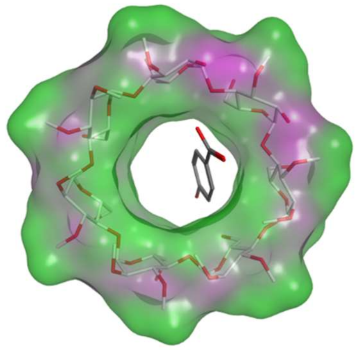

Supplementary Fig. 2. Molecular docking of **M** and **H**.

4. Fourier-Transform IR (FT-IR) spectra of MH

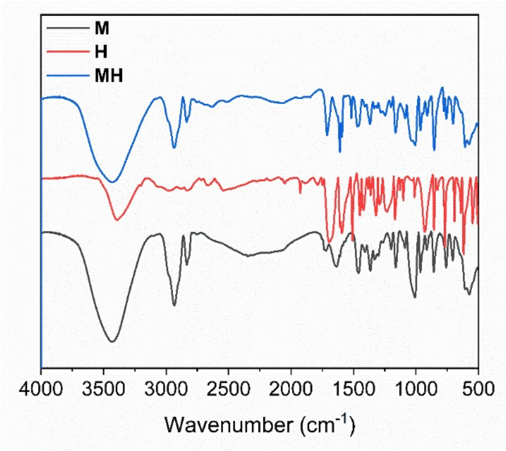

Supplementary Fig. 3. FT-IR spectra of **MH**.

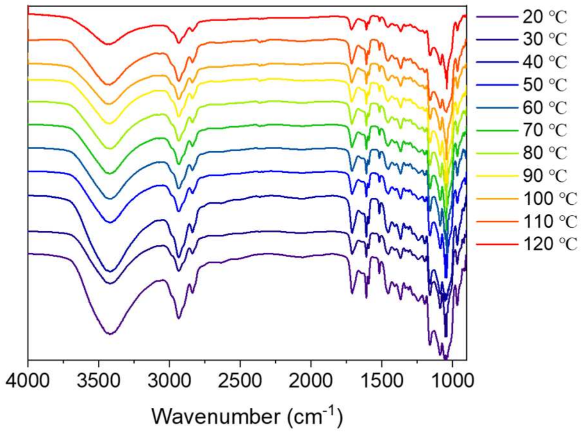

Supplementary Fig. 4. Temperature-dependent FT-IR spectra of **MH** during heating from 20 to 120 ºC.

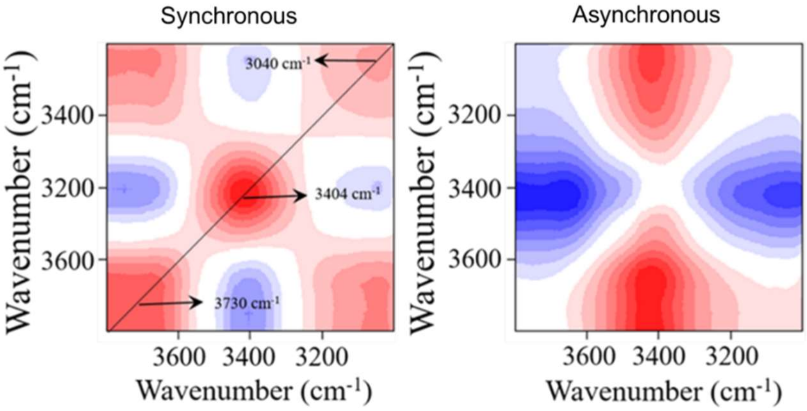

Supplementary Fig. 5. Synchronous and asynchronous maps of **MH** during heating from 20 to 120 ºC.

5. Thermogravimetric Analysis (TGA) of MH

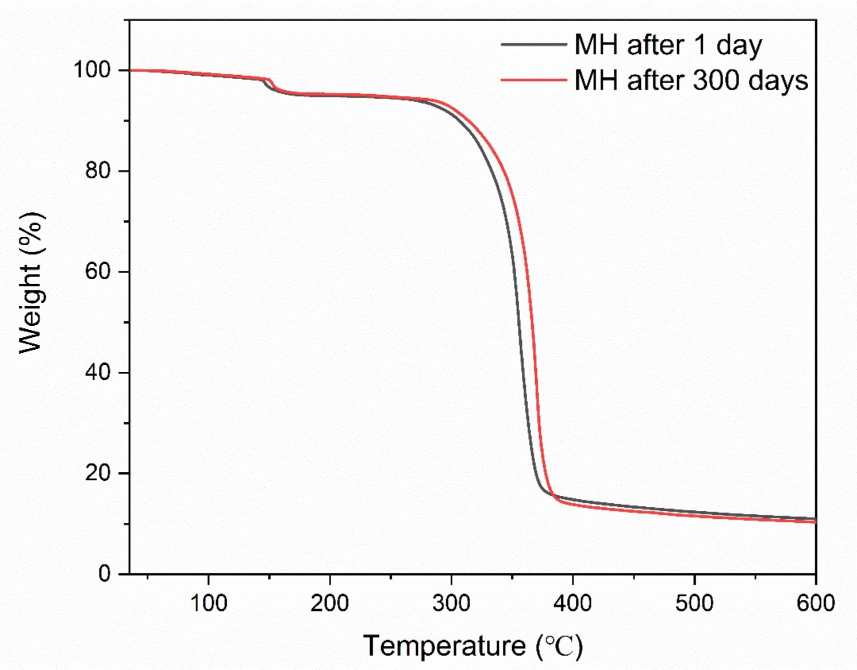

Supplementary Fig. 6. TGA spectra of **MH** stand at different time.

Water molecules in **MH** show good stability in the long-term tests. No dehydration or hygroscopic behavior was observed from **MH** sample that was stored for 300 days, because its TGA curve is similar to that of fresh **MH** sample.

6. Broadband dielectric measurements of MH

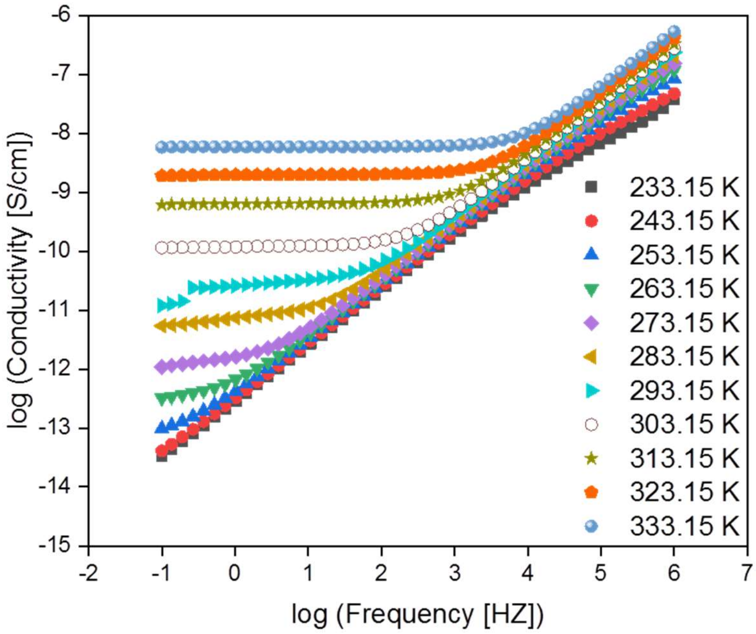

Supplementary Fig. 7. DC conductivity σ_{dc} as a function of frequency for **MH** from – 233.15 to 333.15 K.

7. Single crystal pattern of *para*-hydroxybenzoic acid

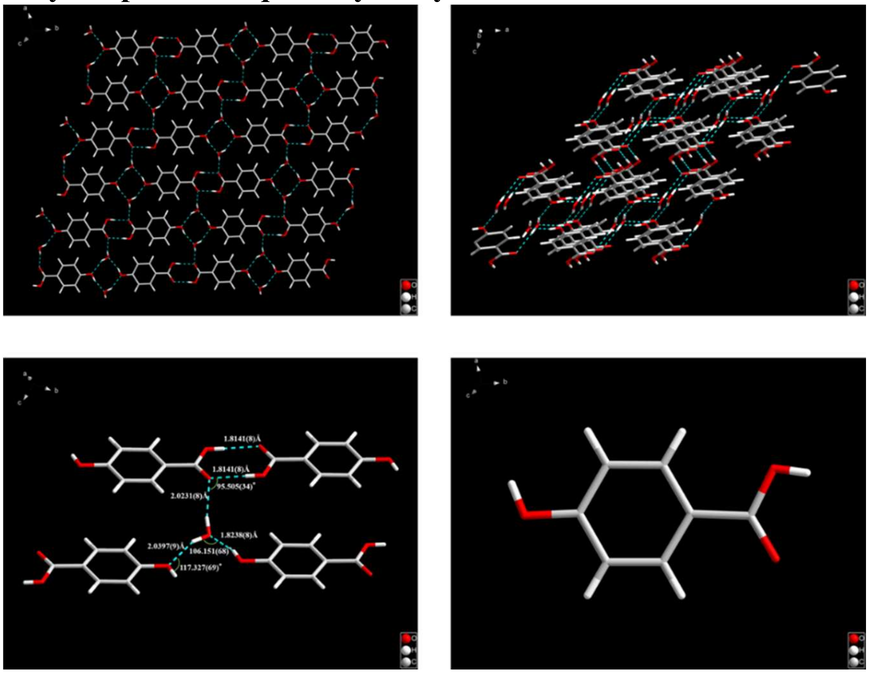

Supplementary Fig. 8. Single crystal pattern of **H** (CCDC: 2239891).

8. Low field nuclear magnetic resonance of MH

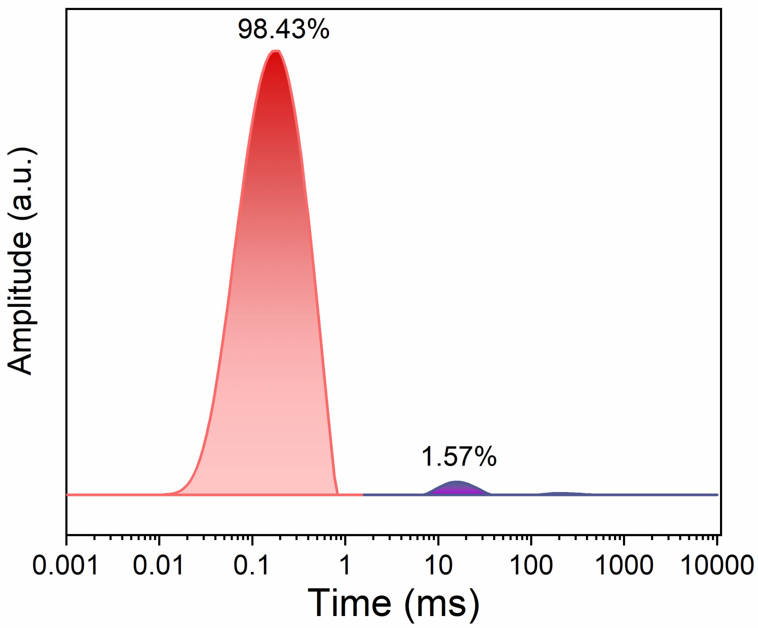

Supplementary Fig. 9. Low field nuclear magnetic resonance of **MH**.

As shown in this Supplementary Fig. 9, it is quite obvious that the majority of water molecules in **MH** belong to bound water, which is consistent with the result from dielectric experiments.

9. Molecular dynamic (MD) simulations

Supplementary Tab. 3. Simulation system.

System	**M**	**H**	Water
1	20	20	0
2	20	20	20
3	20	10	10
4	20	20	5

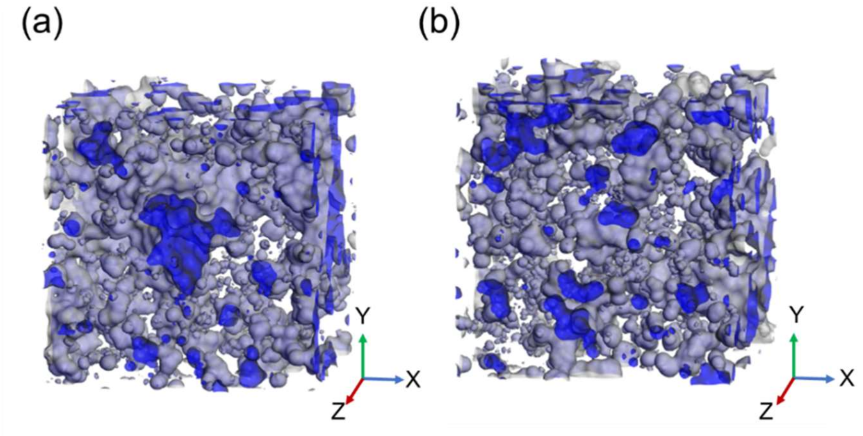

Supplementary Fig. 10. Model of molecular dynamic. (a) 20 **M** + 20 **H** +20 H_2O; (b) 20 **M** + 10 **H** +10 H_2O.

Supplementary Tab. 4. Free volume of complex containing.

System	Volume (Å^3)	Occupied Volume (Å^3)	Free Volume (Å^3)	FFV (%)
1	40319.44	32607.15	7712.29	19.13
2	40076.45	33510.24	6566.21	16.38
3	37401.21	31417.51	5983.70	16.00
4	38662.52	32958.24	5704.28	14.75

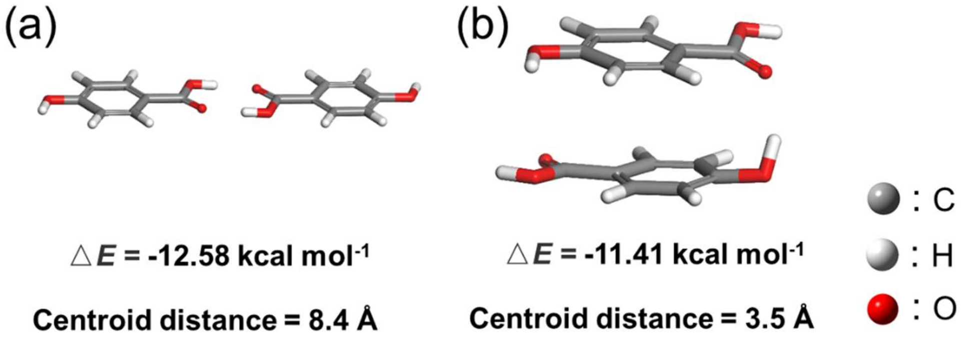

Supplementary Fig. 11. The model of centroid distance and binding energy between **H** and **H**: (a) Centroid distance of 8.4 Å; (b) Centroid distance of 3.5 Å.

Supplementary Tab. 5. Cohesive energy density.

System	Total (J m^{-3})	Van der Waal (J m^{-3})	Electrostatic (J m^{-3})	Other (J m^{-3})
1	4.70×10^8	3.34×10^8	1.24×10^8	1.24×10^7
2	5.20×10^8	3.51×10^8	1.56×10^8	1.30×10^7
3	5.01×10^8	3.51×10^8	1.37×10^8	1.33×10^7
4	5.14×10^8	3.72×10^8	1.28×10^8	1.36×10^7

Those FFV and cohesive energy density show that **MH** with water molecules has high cohesive energy density and low FFV, due to the cross-linking behavior of water molecules.

10. Rheological testing of MH

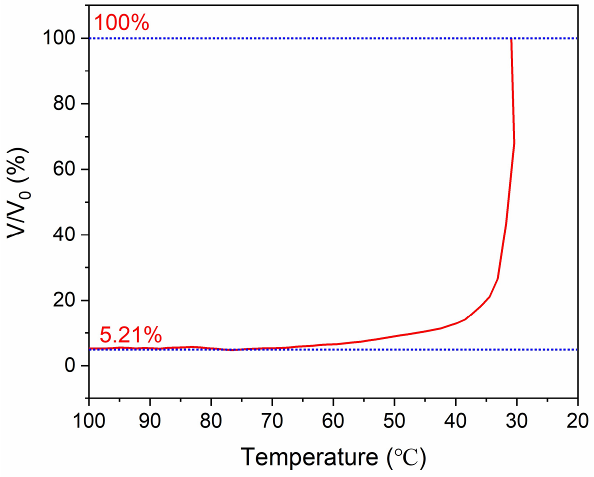

Supplementary Fig. 12. Temperature-dependent viscosity of **MH** (V_0 is the viscosity of **MH** at 30 °C; V is the viscosity of **MH** at different temperatures).

11. Powder X-ray diffraction (PXRD) patterns of MH

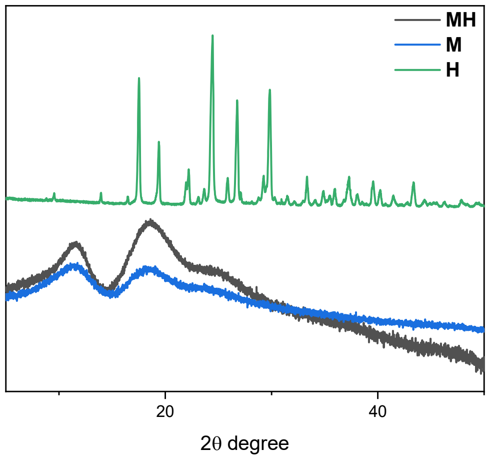

Supplementary Fig. 13. PXRD spectra of **MH**, **M** and **H**.

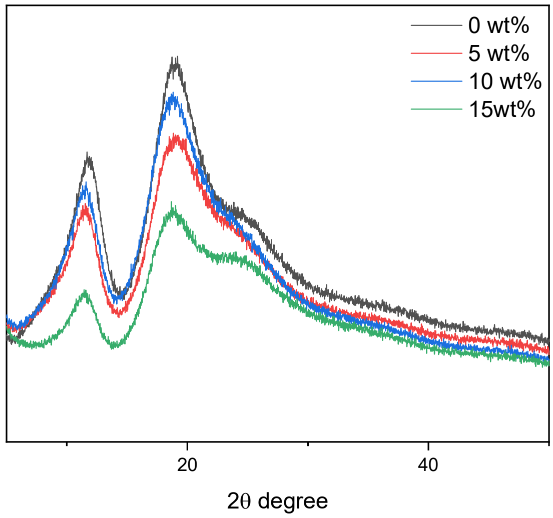

Supplementary Fig. 14. PXRD spectra of **MH** with different water content.

12. Small angle X-ray scattering (SAXS) of MH

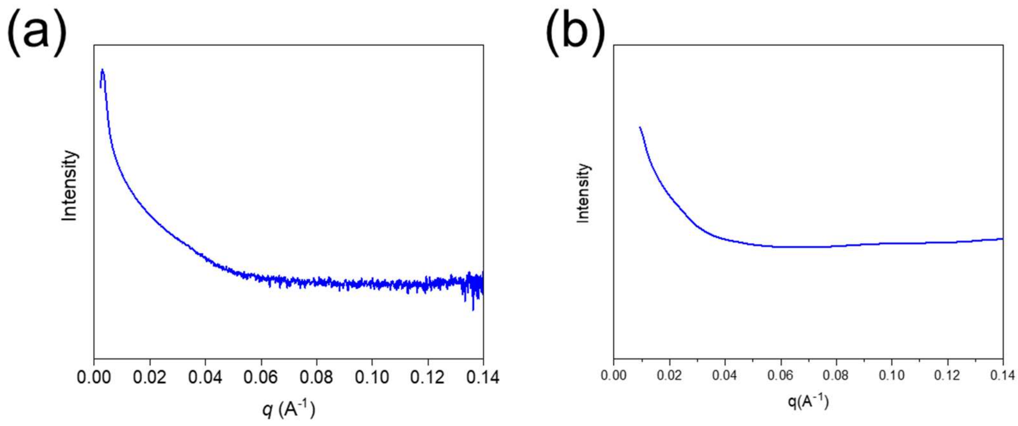

Supplementary Fig. 15. SAXS spectra of **MH** and dried **MH**: (a) **MH**; (b) dried **MH**.

13. Solid-phase ultraviolet spectrum of MH

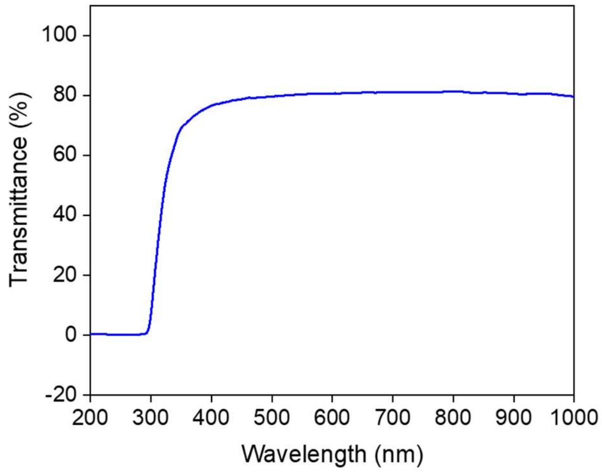

Supplementary Fig. 16. Solid-phase ultraviolet spectrum of **MH**.

14. Differential scanning calorimeter (DSC) of MH

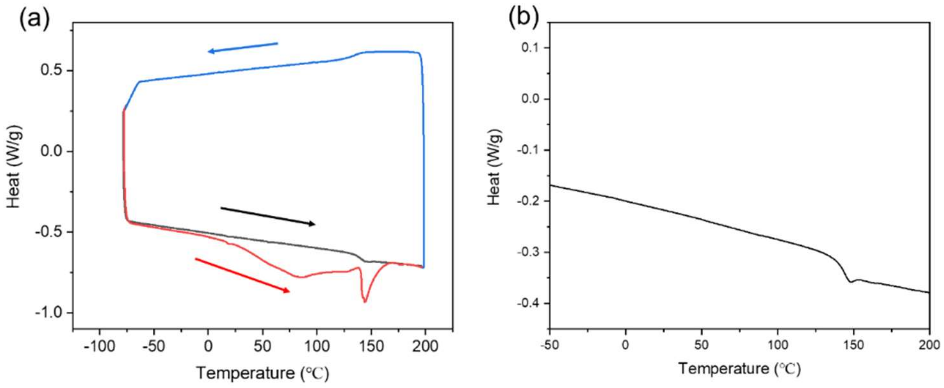

Supplementary Fig. 17. DSC spectra of **MH** and dried **MH**: (a) **MH**; (b) dried **MH**.

Relatively complicated DSC measurements were designed for two main reasons: a) there are water molecules located in the cavity of cyclodextrin: b) because water evaporation method was used, **MH** contains water molecules. Therefore, the role of water should be considered in DSC measurements.

First, the glass sample was heated from –80 °C to 200 °C (the red curve). Then, the glass sample was cooled down from 200 to –80 °C (the blue curve). Finally, the glass sample was heated from –80 to 200 °C (the black curve). The broad peak at around 75 °C can be ascribed to the loss of water molecules located in the cavity of cyclodextrin. The endothermic peak appearing at 150 °C is possibly caused by the loss of bound water.

15. Transmittance of MH

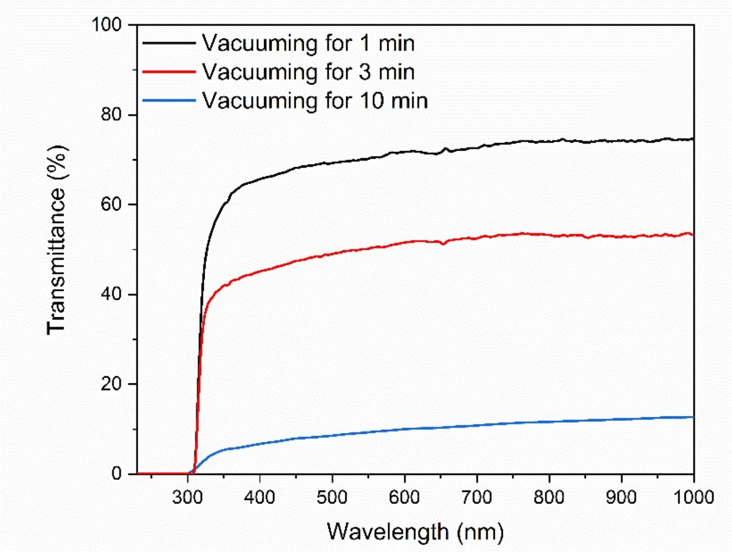

Supplementary Fig. 18. Transmittances of **MH** vacuuming for different time.

Those results indicate that **MH** rapidly becomes opaque due to the loss of water.

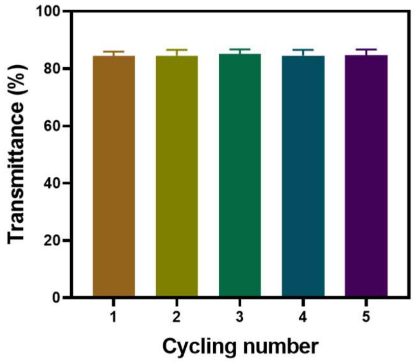

Supplementary Fig. 19. Transmittances of recycled **MH** (route a, 800 nm). Error bars correspond to the standard deviation of 3 measurements for each analysis.

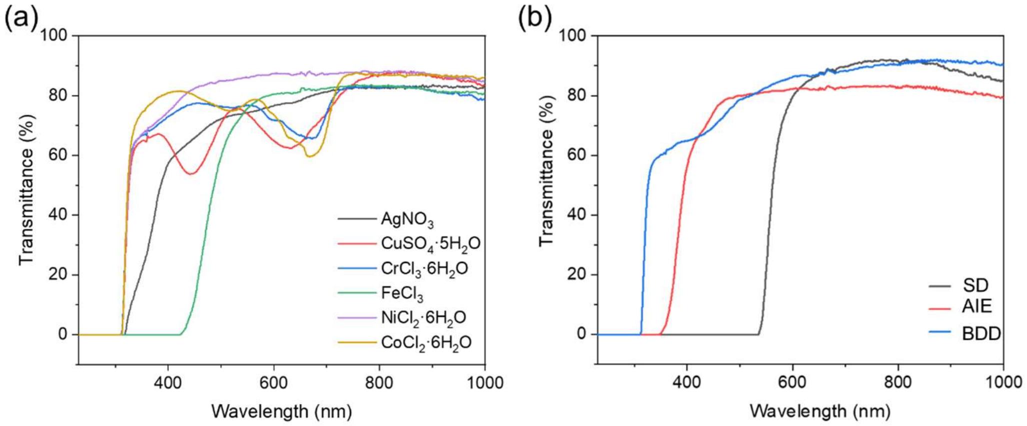

Supplementary Fig. 20. Transmittances of **MH** with additives: (a) Metal ion; (b) organic dye [sudan II (SD); tetrakis(4-hydroxyphenyl)ethylene (AIE); 1,4-bis-(α-cyano-4-methoxystyryl)-2,5-dimethoxybenzene (BDD)].

16. Refractive index measurement of MH

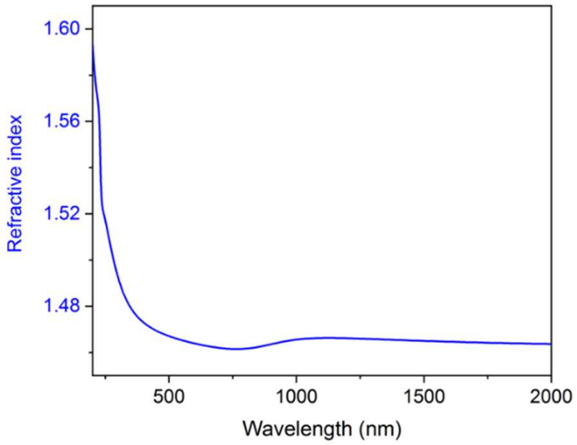

Supplementary Fig. 21. Refractive index of **MH** at different wavelength.

17. Mechanical testing of MH

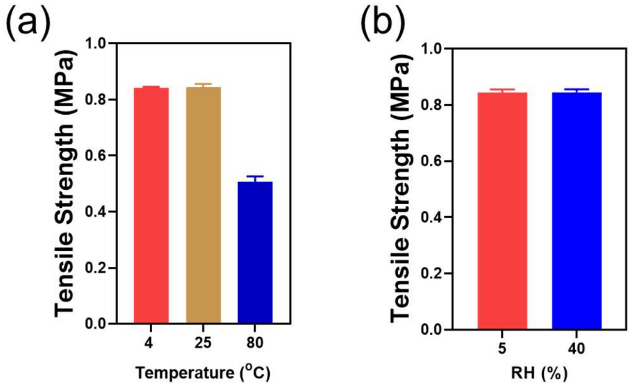

Supplementary Fig. 22. Tensile strength of **MH**: (a) Temperature-dependent tensile stress of **MH** at 30 RH%; (b) Humidity-dependent tensile stress of **MH** at 25 °C. Error bars correspond to the standard deviation of 3 measurements for each analysis.

18. Dynamic mechanic thermal analysis (DMA) of MH

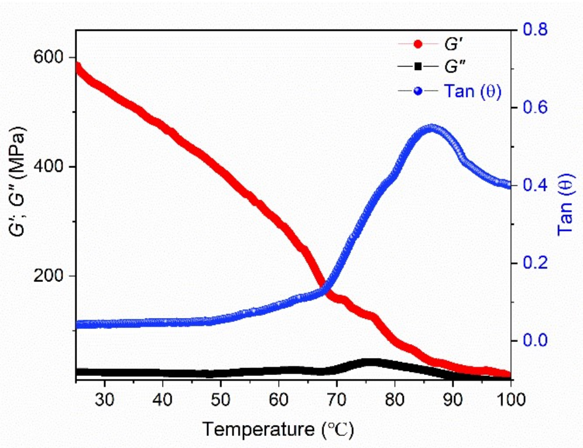

Supplementary Fig. 23. Temperature-dependent of storage modulus (*G′*), loss (*G″*) modulus and loss angle of **MH**.

Glass transition temperature (T_g) was obtained from DMA test, at 86.10 °C.

19. Photos of MH with additives

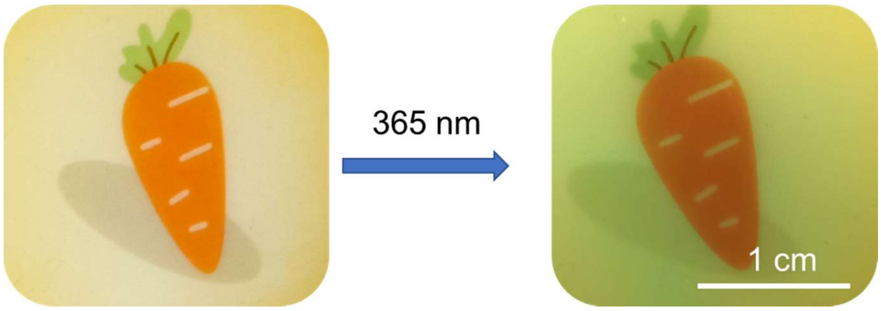

Supplementary Fig. 24. Photos of **MH** with 1,4-bis-(α-cyano-4-methoxystyryl)-2,5-dimethoxybenzene (0.1 wt%).

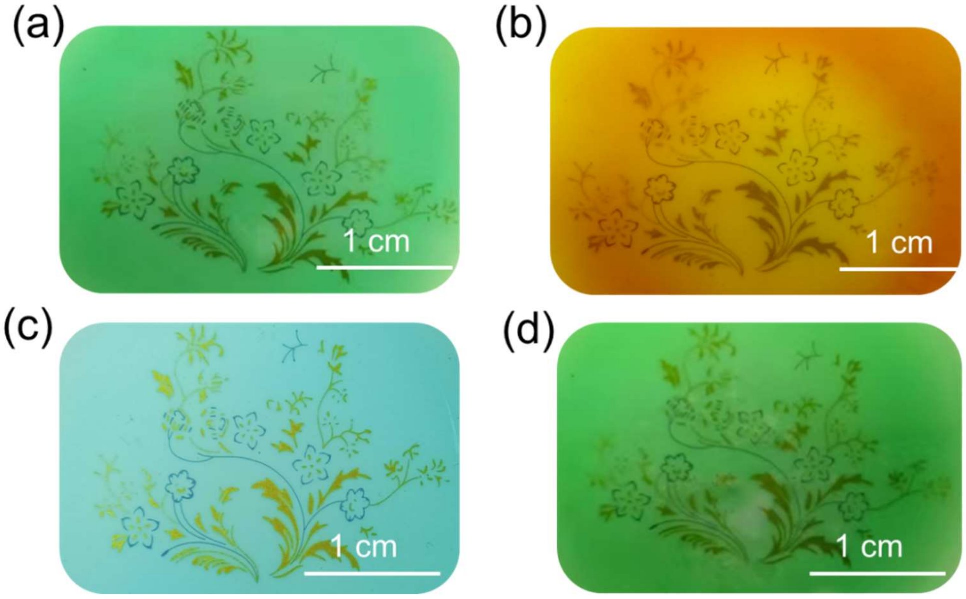

Supplementary Fig. 25. Photos of **MH** with metal ion (10 wt%): (a) $CuSO_4 \cdot 5H_2O$; (b) $FeCl_3$; (c) $CrCl_3 \cdot 6H_2O$; (d) $NiCl_2 \cdot 6H_2O$.

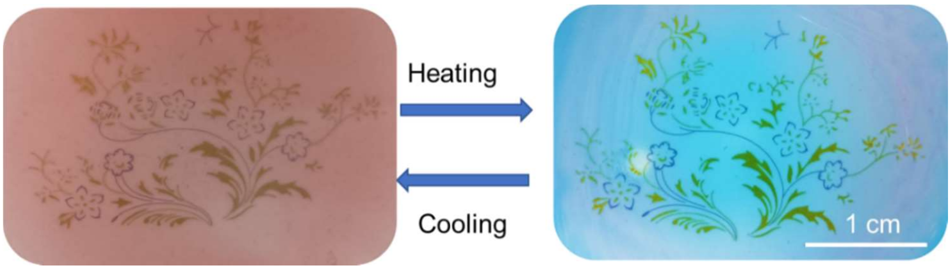

Supplementary Fig. 26. Photos of **MH** with $CoCl_2 \cdot 6H_2O$ (10 wt%).

20. Atomic force microscopy (AFM) of MH

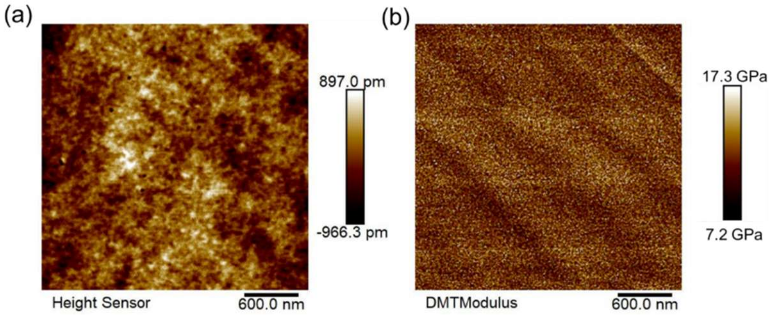

Supplementary Fig. 27. AFM images of **MH** with $CoCl_2 \cdot 6H_2O$: (a) Phase image; (b) modulus.

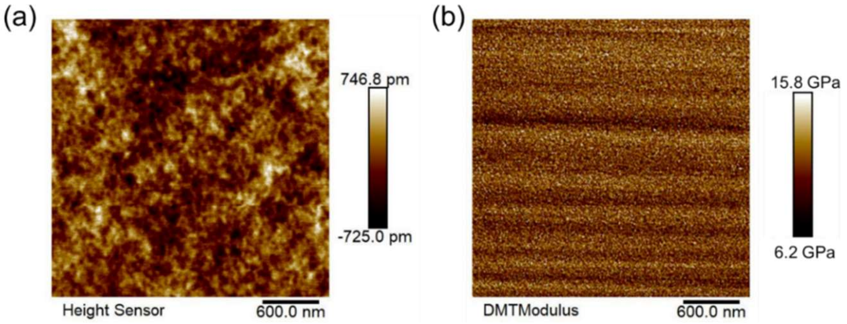

Supplementary Fig. 28. AFM images of **MH** with tetrakis(4-hydroxyphenyl)ethylene: (a) Phase image; (b) modulus.

21. Fluorescence of MH with additives

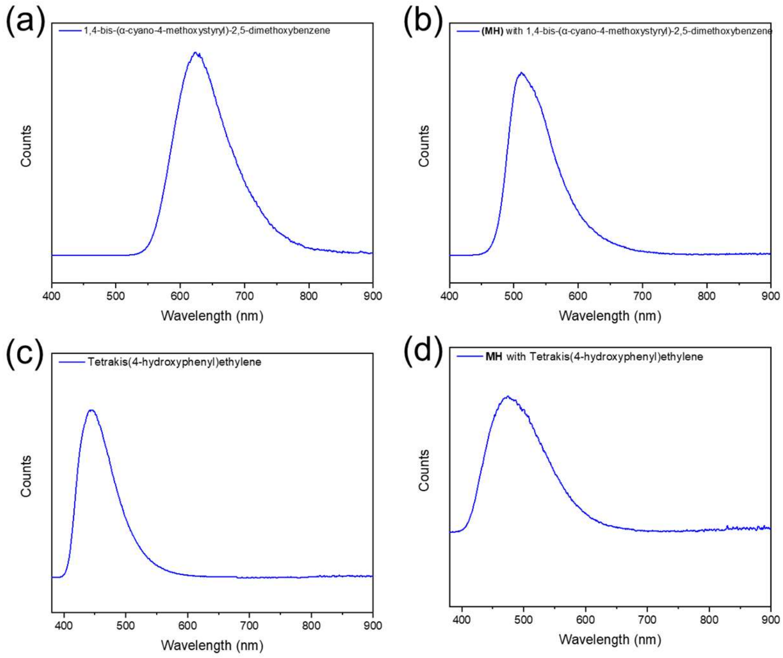

Supplementary Fig. 29. Fluorescence spectra of **MH** with dyes (ex: 365 nm): (a) 1,4-Bis-(α-cyano-4-methoxystyryl)-2,5-dimethoxybenzene; (b) **MH** with 1,4-bis-(α-cyano-4-methoxystyryl)-2,5-dimethoxybenzene; (c) tetrakis(4-hydroxyphenyl)ethylene. (d) **MH** with tetrakis(4-hydroxyphenyl)ethylene.

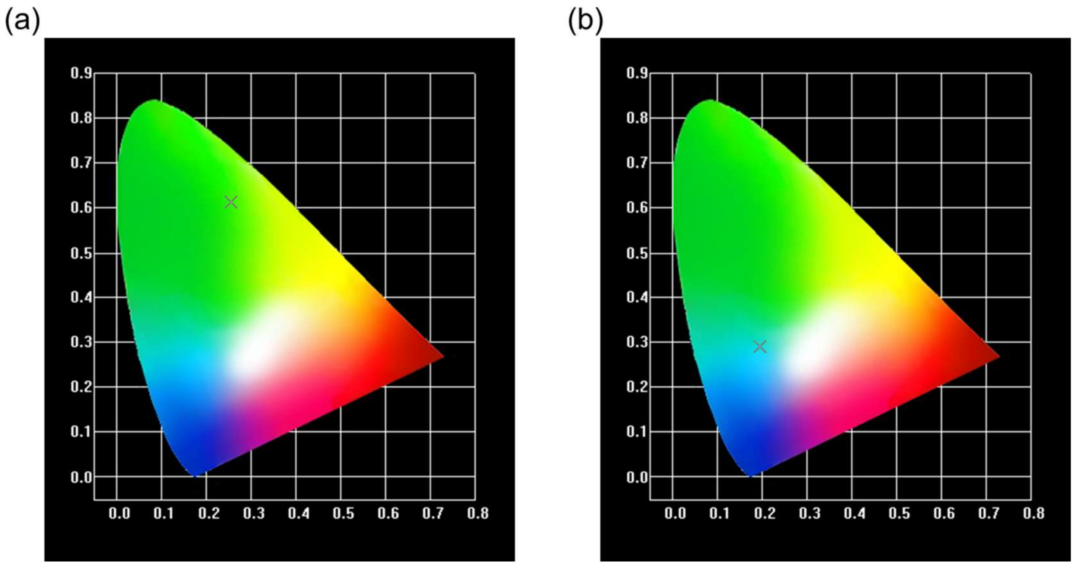

Supplementary Fig. 30. CIE of **MH** with dyes: (a) **MH** with 1,4-bis-(α-cyano-4-methoxystyryl)-2,5-dimethoxybenzene; (b) **MH** with tetrakis(4-hydroxyphenyl)ethylene.

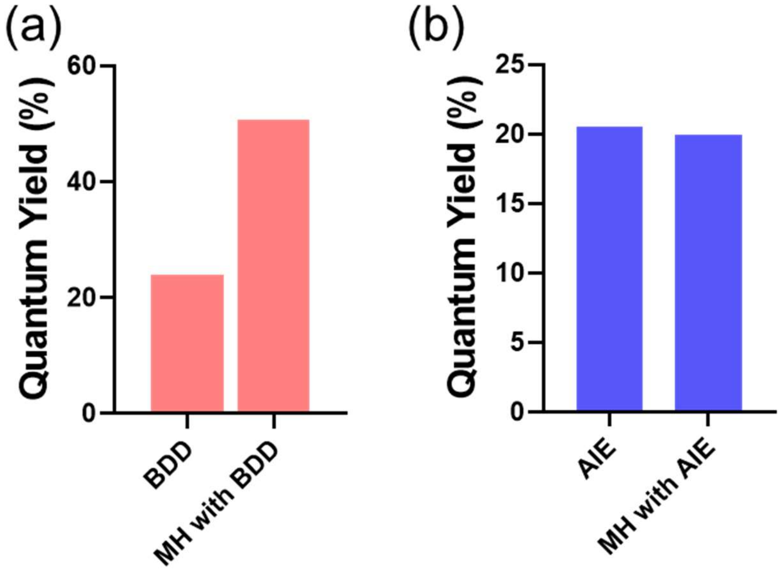

Supplementary Fig. 31. Quantum yield of **MH** with dyes: (a) **MH** with 1,4-bis-(α-cyano-4-methoxystyryl)-2,5-dimethoxybenzene; (b) **MH** with tetrakis(4-hydroxyphenyl)ethylene.

22. NMR spectra of reagent

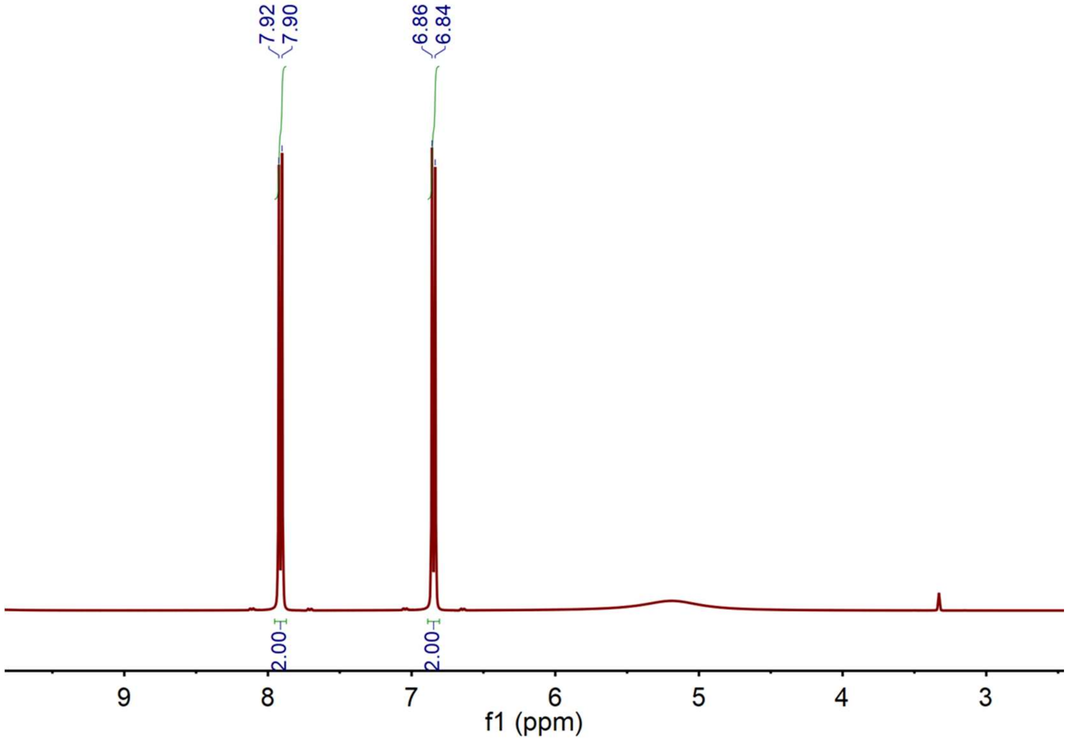

Supplementary Fig. 32. ^{1}H NMR spectrum of **H**.

^{1}H NMR (400 MHz, CD_3OD, 25 °C) δ 7.90–7.92 (d, *J* = 8.0 Hz, 2H), 6.86–6.84 (d, *J* = 8.0 Hz, 2H).

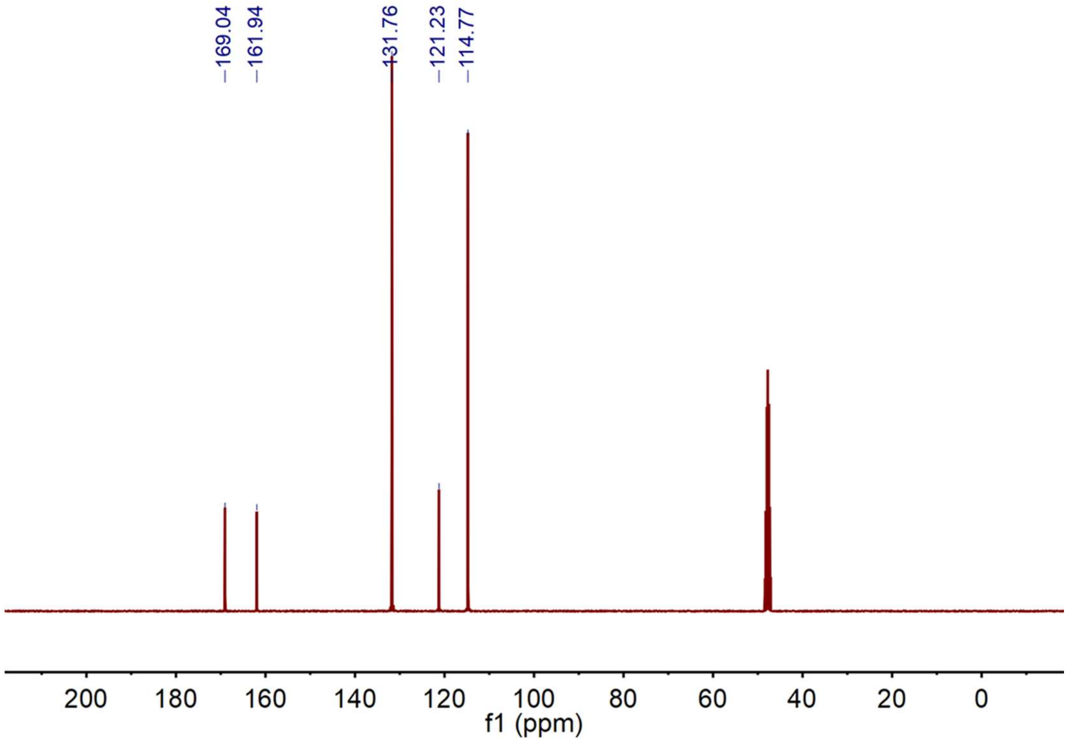

Supplementary Fig. 33. ^{13}C NMR spectrum of **H**.

^{13}C NMR (400 MHz, CD_3OD, 25 °C) δ 169.04, 161.94, 131.76, 121.23, 114.77.

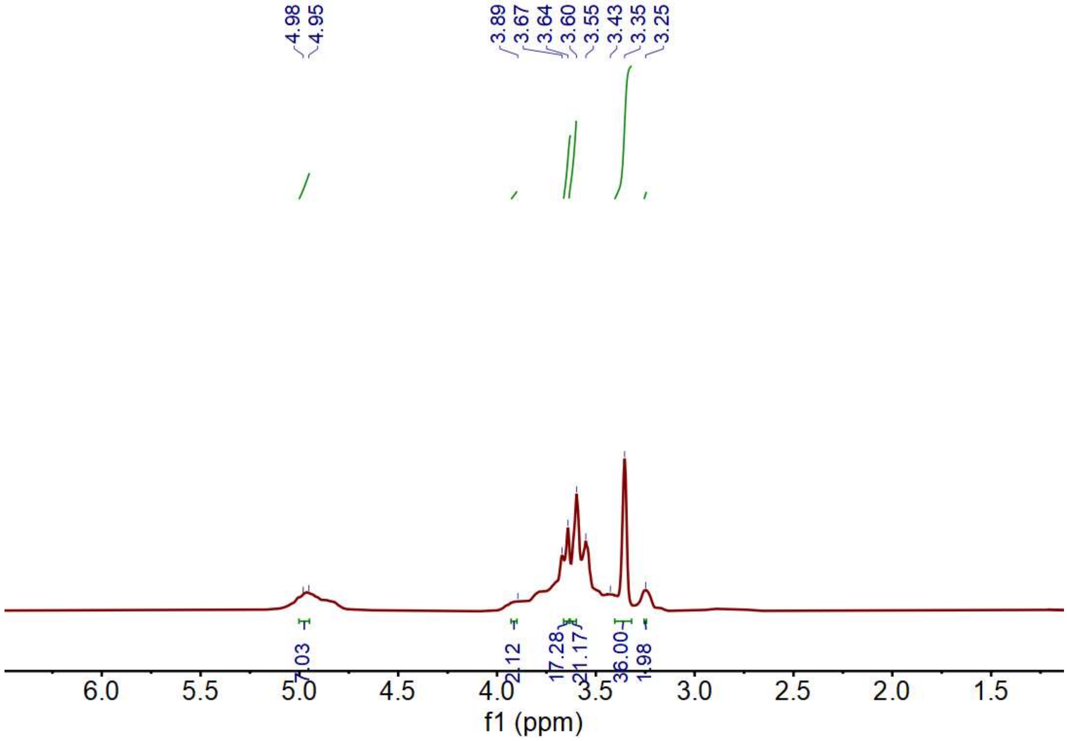

Supplementary Fig. 34. ^{1}H NMR spectrum of **M**.
^{1}H NMR (400 MHz, CD_3Cl, 25 °C) δ 4.98–4.95 (m, 7H), 3.89 (m, 2H), 3.66–3.63 (m, 17H), 3.64–3.60 (m, 21H), 3.35 (m, 36H), 3.25 (m, 2H).

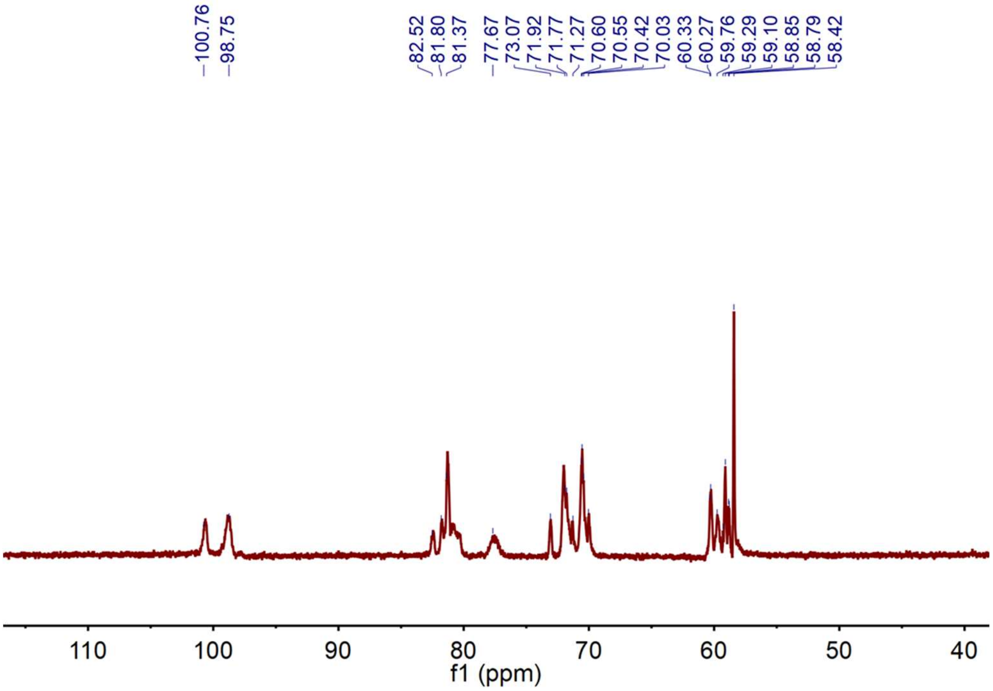

Supplementary Fig. 35. ^{13}C NMR spectrum of **M**.^{13}C NMR (400 MHz, D_2O, NaCl:12 mg/mL, 25 °C,) δ 100.76, 98.75, 82.52, 81.8, 81.37, 77.67, 73.07, 71.92, 71.77, 71.27, 70.60, 70.55, 70.42, 70.03, 60.33, 60.27, 59.76, 59.29, 59.10, 58.85, 58.79, 58.42.

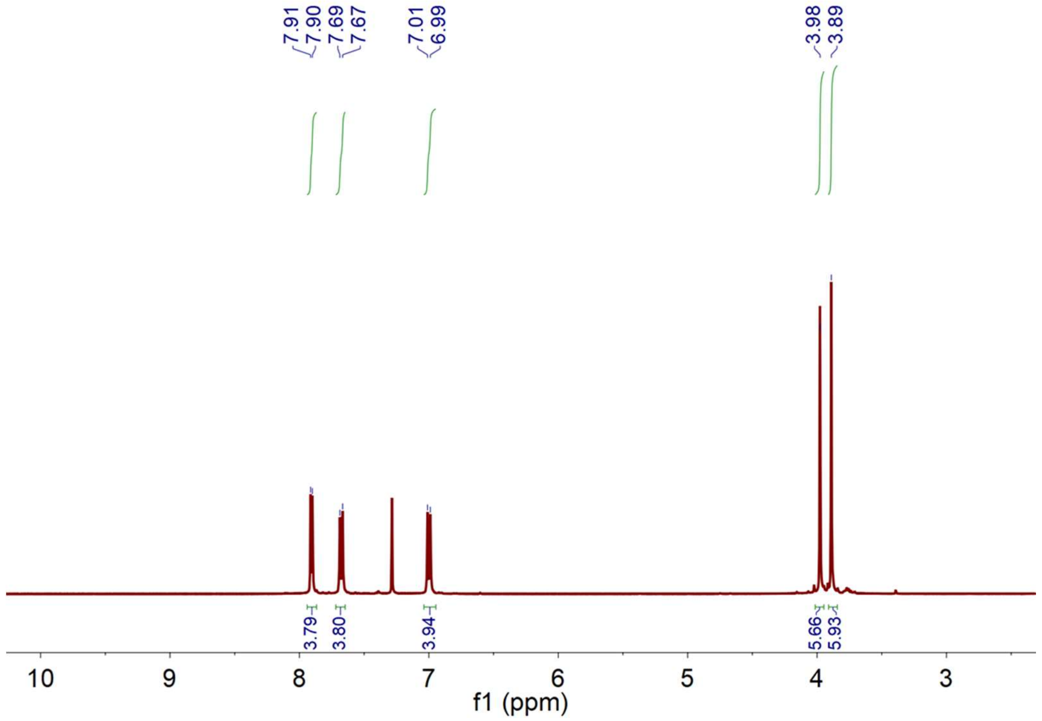

Supplementary Fig. 36. ^{1}H NMR spectrum of 1,4-bis-(α-cyano-4-methoxystyryl)-2,5-dimethoxybenzene.

^{1}H NMR (400 MHz, $CDCl_3$, 25 °C) δ: 7.90–7.91 (d, J = 4.0 Hz, 4H), 7.67–7.69 (d, J = 8.0 Hz, 4H), 6.99–7.01 (d, J = 8.0 Hz, 4H), 3.98 (s, 6H), 3.89 (s, 6H).

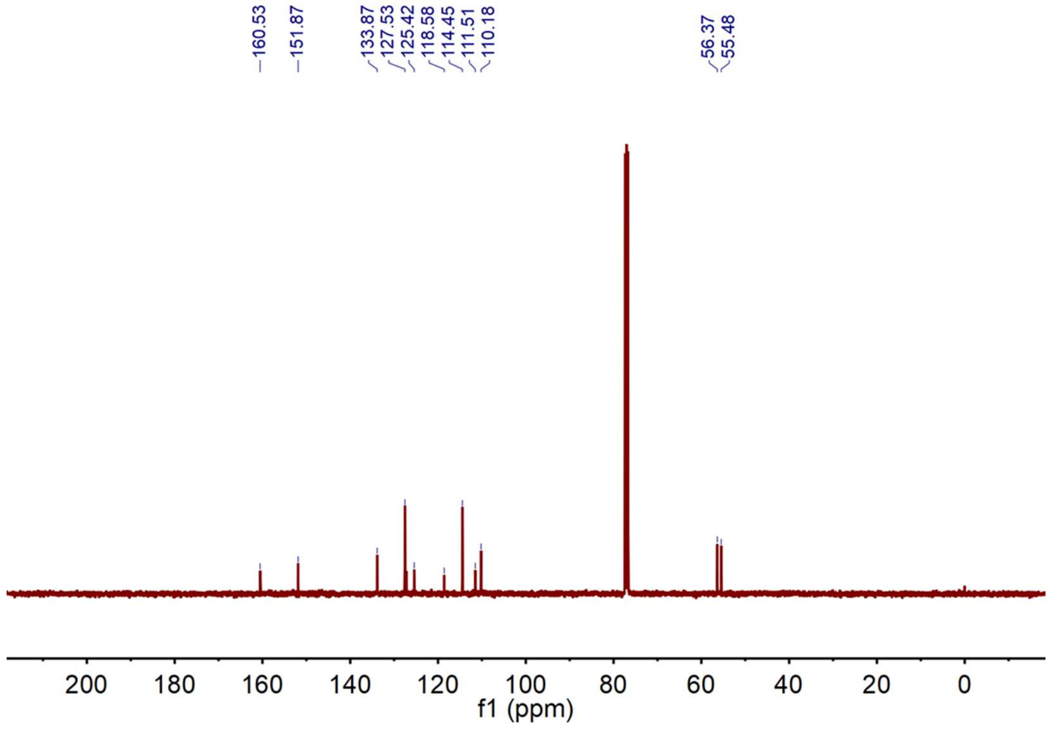

Supplementary Fig. 37. ^{13}C NMR spectrum of 1,4-bis-(α-cyano-4-methoxystyryl)-2,5-dimethoxybenzene.

^{13}C NMR (400 MHz, $CDCl_3$, 25 °C) δ ppm: 160.53, 151.87, 133.87, 127.53, 125.42, 118.58, 114.45, 111.51, 110.18, 56.37, 55.48.

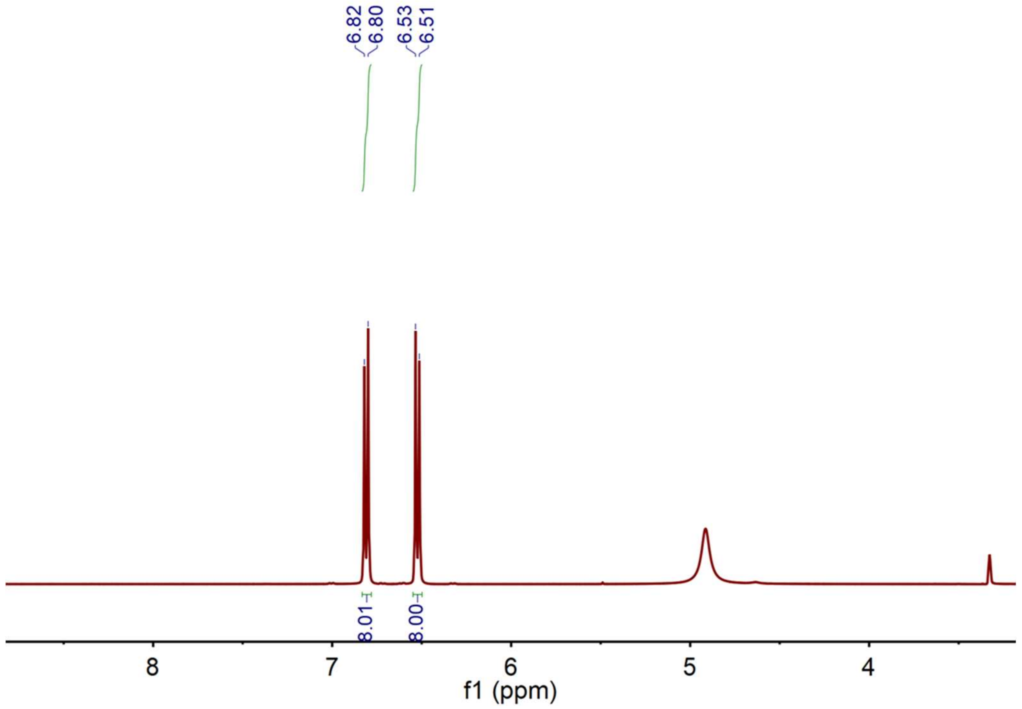

Supplementary Fig. 38. ^{1}H NMR spectrum of tetrakis(4-hydroxyphenyl)ethylene. ^{1}H NMR (400 MHz, CD_3OD, 25 °C) δ 6.80–6.82 (d, J = 8.0 Hz, 8H), 6.51–6.53 (d, J = 8.0 Hz, 8H).

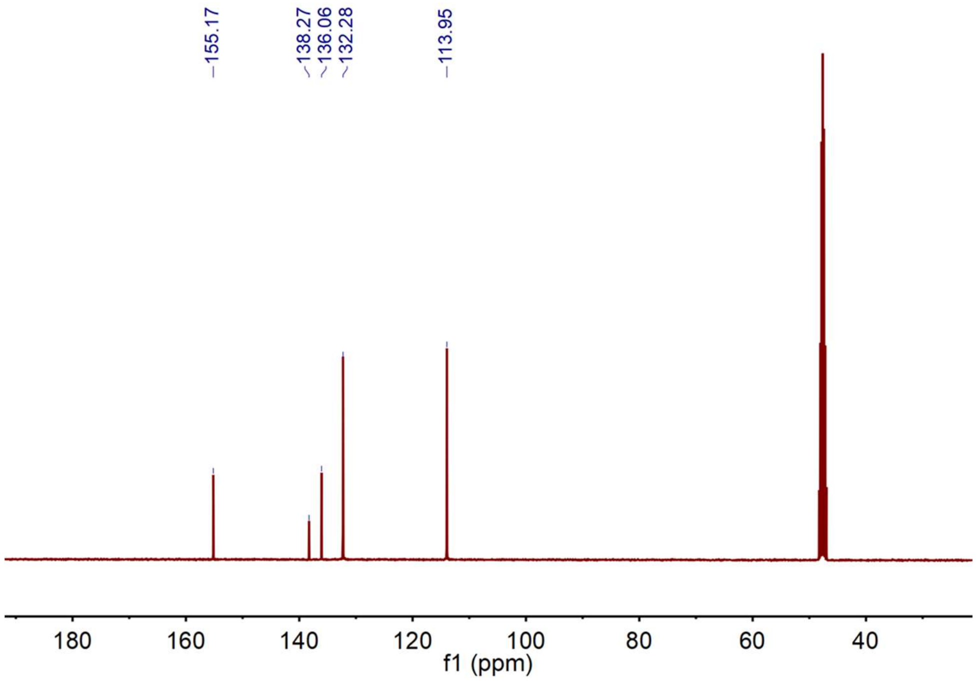

Supplementary Fig. 39. ^{13}C NMR spectrum of tetrakis(4-hydroxyphenyl)ethylene. ^{13}C NMR (400 MHz, CD_3OD, 25 °C) δ 155.17, 138.27, 136.06, 132.28, 113.95.

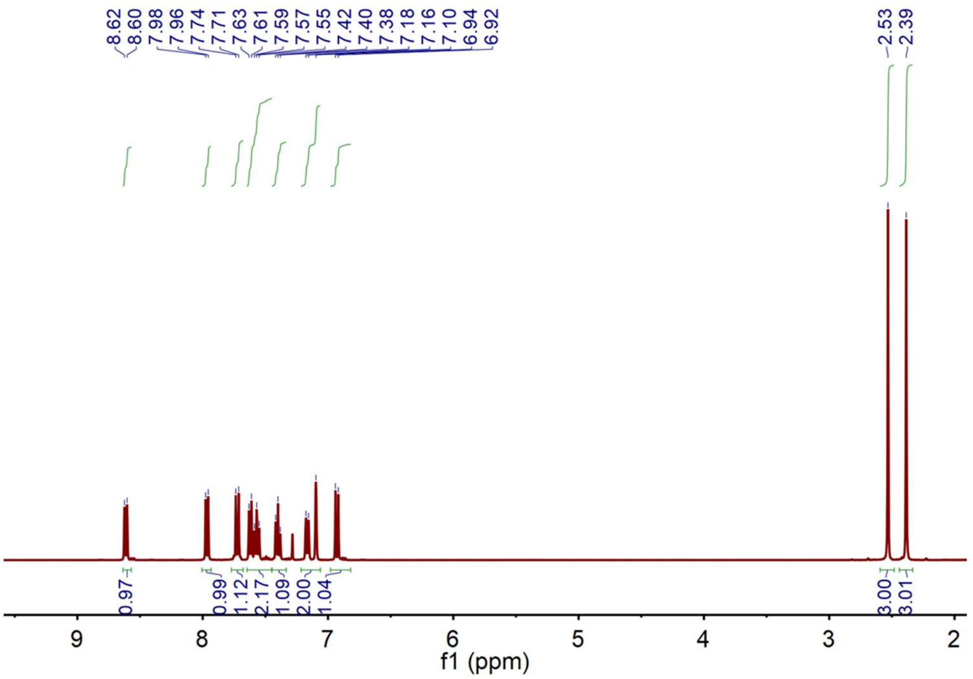

Supplementary Fig. 40. ^{1}H NMR spectrum of Sudan II.

^{1}H NMR (400 MHz, $CDCl_3$, 25 °C) δ 8.60–8.62 (d, *J* = 8.0 Hz, 1H), 7.96–7.98 (d, *J* = 8.0 Hz, 1H), 7.71–7.74 (d, *J* =12.0 Hz, 1H), 7.55–7.63 (m, 2H), 7.38–7.42 (t, *J* = 16 Hz, 1H), 7.10–7.18 (m, 2H), 6.92–6.94 (m, 1H), 2.53 (s, 3H), 2. 39 (s, 3H).

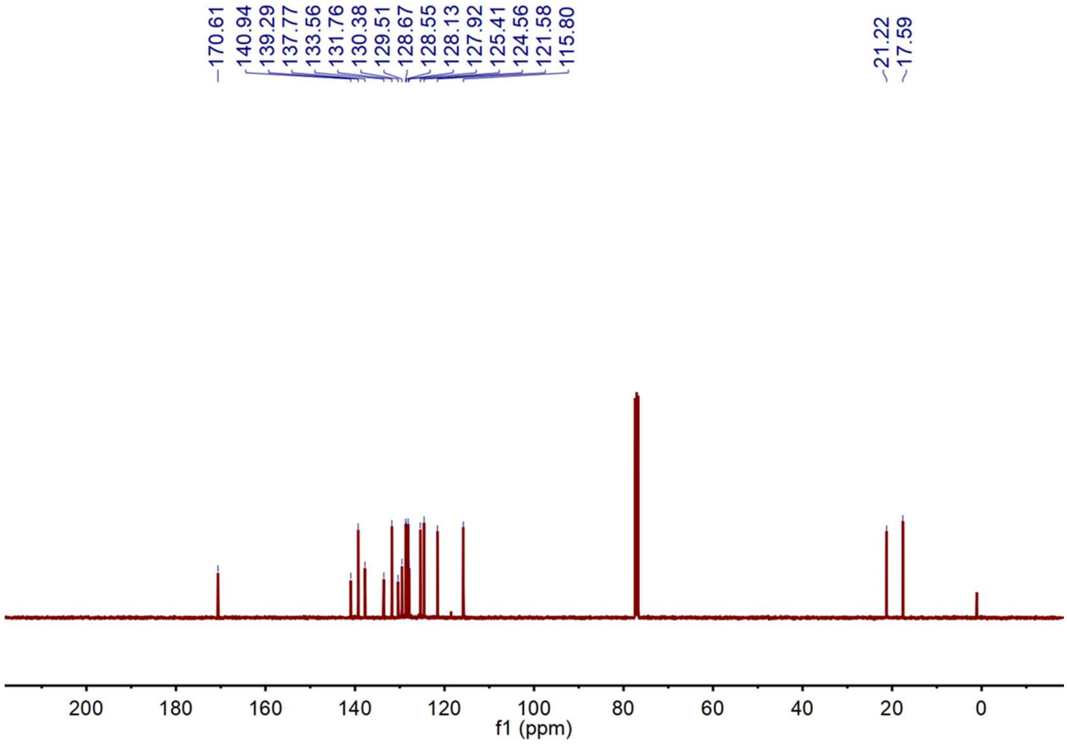

Supplementary Fig. 41. ^{13}C NMR spectrum of Sudan II.

^{13}C NMR (400 MHz, $CDCl_3$, 25 °C) δ 170.62, 140.94, 139.29, 137.77, 133.56, 131.76, 130.38, 129.51, 128.67, 128.55, 128.13, 127.92, 125.41, 124.56, 121.58, 115.80, 21.22, 17.59.
